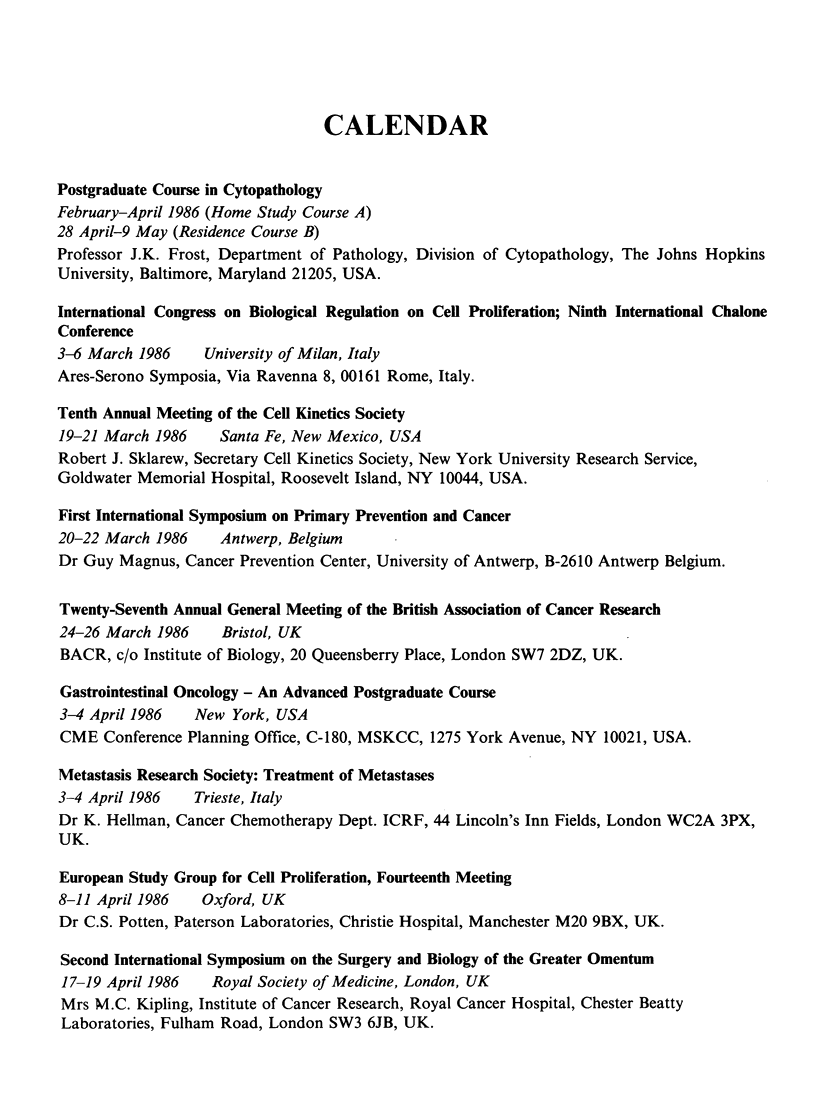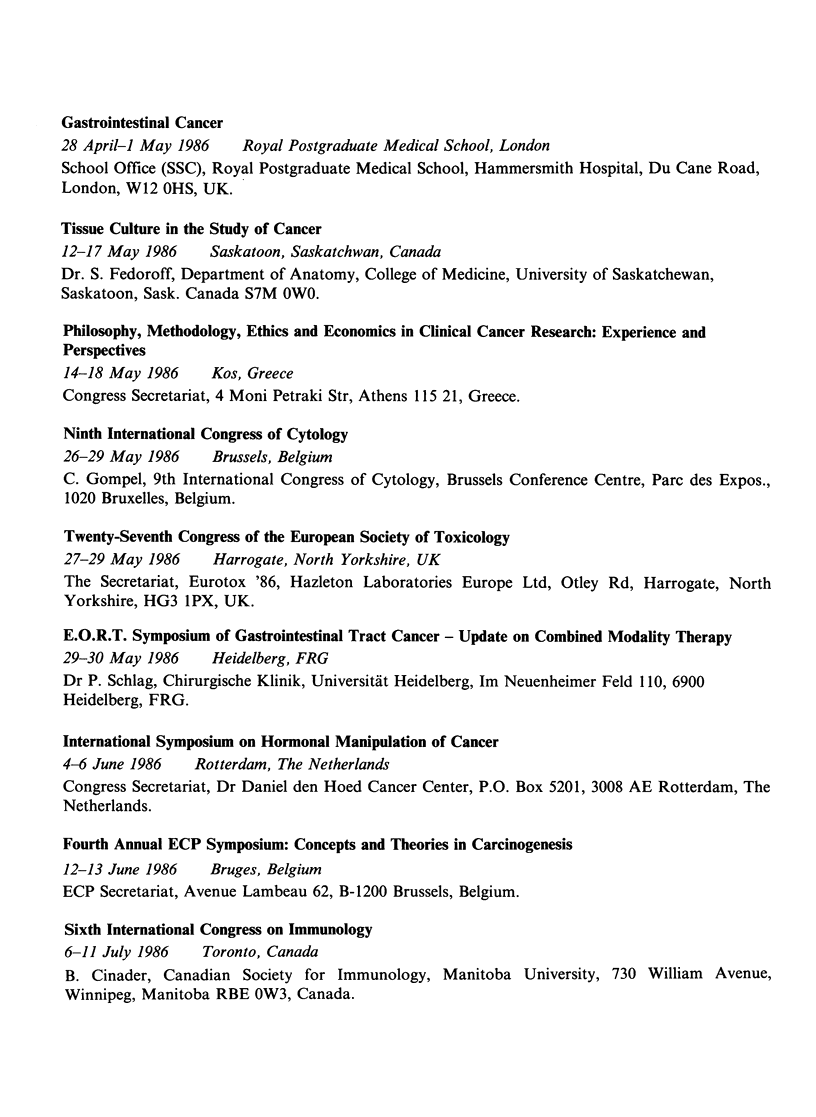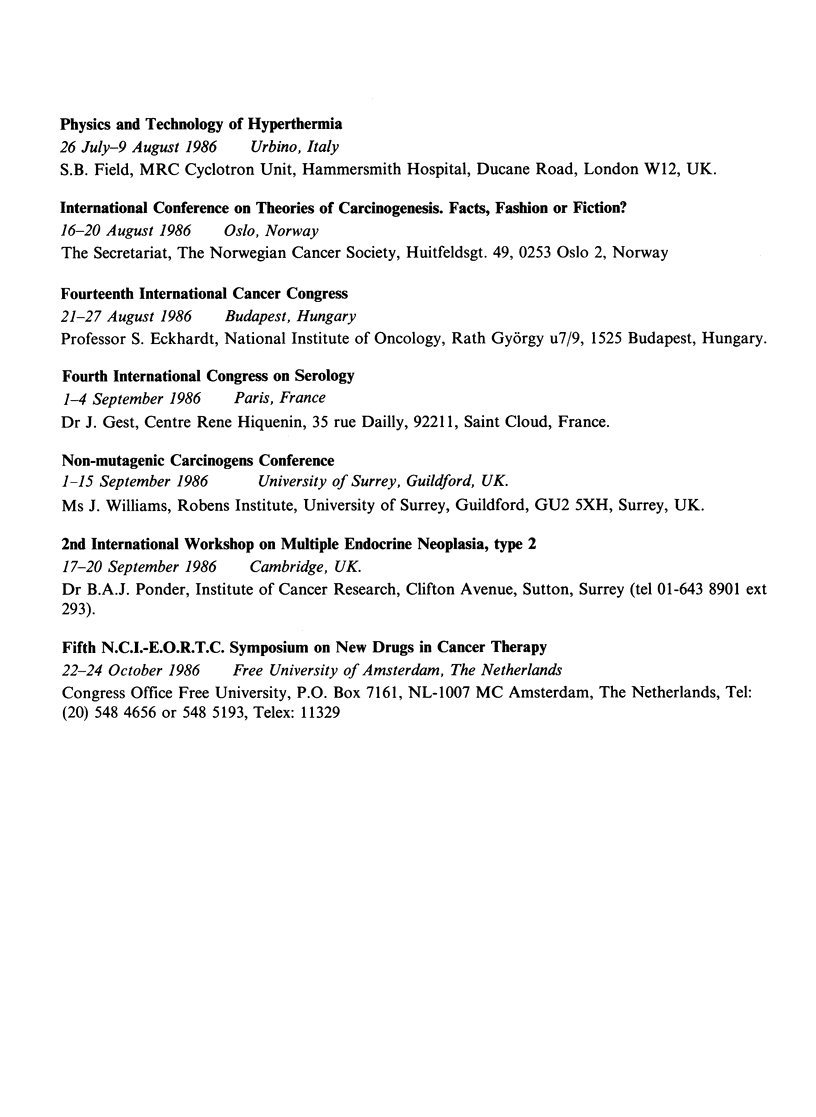# Calendar

**Published:** 1986-03

**Authors:** 


### CALENDAR

**Postgraduate Course in Cytopathology**
*February–April 1986 (Home Study Course A)*
*28 April–9 May (Residence Course B)*
Professor J.K. Frost, Department of Pathology, Division of Cytopathology, The Johns Hopkins University, Baltimore, Maryland 21205, USA.

**International Congress on Biological Regulation on Cell Proliferation; Ninth International Chalone Conference**
*3–6 March 1986  University of Milan, Italy*
Ares-Serono Symposia, Via Ravenna 8, 00161 Rome, Italy.

**Tenth Annual Meeting of the Cell Kinetics Society**
*19–21 March 1986  Santa Fe, New Mexico, USA*
Robert J. Sklarew, Secretary Cell Kinetics Society, New York University Research Service, Goldwater Memorial Hospital, Roosevelt Island, NY 10044, USA.

**First International Symposium on Primary Prevention and Cancer**
*20–22 March 1986  Antwerp, Belgium*
Dr Guy Magnus, Cancer Prevention Center, University of Antwerp, B-2610 Antwerp Belgium.

**Twenty-Seventh Annual General Meeting of the British Association of Cancer Research**
*24–26 March 1986  Bristol, UK*
BACR, c/o Institute of Biology, 20 Queensberry Place, London SW7 2DZ, UK.

**Gastrointestinal Oncology – An Advanced Postgraduate Course**
*3–4 April 1986  New York, USA*
CME Conference Planning Office, C-180, MSKCC, 1275 York Avenue, NY 10021, USA.

**Metastasis Research Society: Treatment of Metastases**
*3–4 April 1986  Trieste, Italy*
Dr K. Hellman, Cancer Chemotherapy Dept. ICRF, 44 Lincoln's Inn Fields, London WC2A 3PX, UK.

**European Study Group for Cell Proliferation, Fourteenth Meeting**
*8–11 April 1986  Oxford, UK*
Dr C.S. Potten, Paterson Laboratories, Christie Hospital, Manchester M20 9BX, UK.

**Second International Symposium on the Surgery and Biology of the Greater Omentum**
*17–19 April 1986  Royal Society of Medicine, London, UK*
Mrs M.C. Kipling, Institute of Cancer Research, Royal Cancer Hospital, Chester Beatty Laboratories, Fulham Road, London SW3 6JB, UK.

**Gastrointestinal Cancer**

*28 April–1 May 1986*   *Royal Postgraduate Medical School, London*

School Office (SSC), Royal Postgraduate Medical School, Hammersmith Hospital, Du Cane Road, London, W12 0HS, UK.

**Tissue Culture in the Study of Cancer**

*12–17 May 1986*   *Saskatoon, Saskatchwan, Canada*

Dr. S. Fedoroff, Department of Anatomy, College of Medicine, University of Saskatchewan, Saskatoon, Sask. Canada S7M 0W0.

**Philosophy, Methodology, Ethics and Economics in Clinical Cancer Research: Experience and Perspectives**

*14–18 May 1986*   *Kos, Greece*

Congress Secretariat, 4 Moni Petraki Str, Athens 115 21, Greece.

**Ninth International Congress of Cytology**

*26–29 May 1986*   *Brussels, Belgium*

C. Gompel, 9th International Congress of Cytology, Brussels Conference Centre, Parc des Expos., 1020 Bruxelles, Belgium.

**Twenty-Seventh Congress of the European Society of Toxicology**

*27–29 May 1986*   *Harrogate, North Yorkshire, UK*

The Secretariat, Eurotox '86, Hazleton Laboratories Europe Ltd, Otley Rd, Harrogate, North Yorkshire, HG3 1PX, UK.

**E.O.R.T. Symposium of Gastrointestinal Tract Cancer – Update on Combined Modality Therapy**

*29–30 May 1986*   *Heidelberg, FRG*

Dr P. Schlag, Chirurgische Klinik, Universität Heidelberg, Im Neuenheimer Feld 110, 6900 Heidelberg, FRG.

**International Symposium on Hormonal Manipulation of Cancer**

*4–6 June 1986*   *Rotterdam, The Netherlands*

Congress Secretariat, Dr Daniel den Hoed Cancer Center, P.O. Box 5201, 3008 AE Rotterdam, The Netherlands.

**Fourth Annual ECP Symposium: Concepts and Theories in Carcinogenesis**

*12–13 June 1986*   *Bruges, Belgium*

ECP Secretariat, Avenue Lambeau 62, B-1200 Brussels, Belgium.

**Sixth International Congress on Immunology**

*6–11 July 1986*   *Toronto, Canada*

B. Cinader, Canadian Society for Immunology, Manitoba University, 730 William Avenue, Winnipeg, Manitoba RBE 0W3, Canada.

**Physics and Technology of Hyperthermia**
*26 July–9 August 1986* *Urbino, Italy*
S.B. Field, MRC Cyclotron Unit, Hammersmith Hospital, Ducane Road, London W12, UK.

**International Conference on Theories of Carcinogenesis. Facts, Fashion or Fiction?**
*16–20 August 1986* *Oslo, Norway*
The Secretariat, The Norwegian Cancer Society, Huitfeldsgt. 49, 0253 Oslo 2, Norway

**Fourteenth International Cancer Congress**
*21–27 August 1986* *Budapest, Hungary*
Professor S. Eckhardt, National Institute of Oncology, Rath György u7/9, 1525 Budapest, Hungary.

**Fourth International Congress on Serology**
*1–4 September 1986* *Paris, France*
Dr J. Gest, Centre Rene Hiquenin, 35 rue Dailly, 92211, Saint Cloud, France.

**Non-mutagenic Carcinogens Conference**
*1–15 September 1986* *University of Surrey, Guildford, UK.*
Ms J. Williams, Robens Institute, University of Surrey, Guildford, GU2 5XH, Surrey, UK.

**2nd International Workshop on Multiple Endocrine Neoplasia, type 2**
*17–20 September 1986* *Cambridge, UK.*
Dr B.A.J. Ponder, Institute of Cancer Research, Clifton Avenue, Sutton, Surrey (tel 01-643 8901 ext 293).

**Fifth N.C.I.-E.O.R.T.C. Symposium on New Drugs in Cancer Therapy**
*22–24 October 1986* *Free University of Amsterdam, The Netherlands*
Congress Office Free University, P.O. Box 7161, NL-1007 MC Amsterdam, The Netherlands, Tel: (20) 548 4656 or 548 5193, Telex: 11329